# Predicting Death After Thrombectomy in the Treatment of Acute Stroke

**DOI:** 10.3389/fsurg.2020.00016

**Published:** 2020-04-08

**Authors:** Al-Wala Awad, Craig Kilburg, Vijay M. Ravindra, Jonathan Scoville, Evan Joyce, Ramesh Grandhi, Philipp Taussky

**Affiliations:** Department of Neurosurgery, Clinical Neurosciences Center, University of Utah, Salt Lake City, UT, United States

**Keywords:** emergent thrombectomy, death, stroke, cerebral angiography, endovascular

## Abstract

**Introduction:** Treatments for acute stroke have significantly improved in the past decade, with emergent thrombectomy emerging as the standard of care. Despite these advancements, death after successful thrombectomy continues to pose a significant problem. Identifying patients least likely to benefit from thrombectomy would improve use of a limited resource and management of patient expectations.

**Method:** We retrospectively reviewed the medical records of patients who underwent emergent thrombectomy of either anterior or posterior circulation strokes between January 2012 and January 2017. Relevant patient clinical data was collected and analyzed in a multivariable regression with a primary outcome of death at 90 days.

**Results:** A total of 134 patients underwent emergent endovascular thrombectomy during the study period; sufficient clinical data was available in 111 of the them. Of these, 42 patients died during the 90 day post-procedural period and 69 patients survived this period. The mean NIHSS score at presentation was 14.9 in surviving patients and 19.6 in non-surviving patients (*p* < 0.002). Surviving patients were less likely to have a history of cancer (4.4% vs. 26.2%, *p* < 0.002), achieved higher rates of revascularization (78.3% vs. 50.0%, *p* < 0.003), had a lower rate of hemorrhagic conversion (21.7% vs. 47.6%, *p* < 0.004), and experienced fewer technical complications during their treatment (7.4% vs. 26.2%, *p* < 0.01). Overall, there were 16 intraprocedural complications and no procedural deaths.

**Conclusion:** As emergent thrombectomy for the treatment of acute stroke becomes more prevalent, appropriate patient selection will be crucial in the utilization of a limited and costly intervention. Death within 90 days after thrombectomy appears to be more prevalent among patients with higher NIHSS at presentation, those with postprocedural hemorrhage or intraprocedural complications, and those with a history of cancer.

## Introduction

Stroke continues to be the leading cause of disability and preventable death in the United States (1), with the cost of medical care for patients with stroke in the United States at over $30 billion annually. Over 85% of strokes are ischemic in nature; it is estimated that 11% of these patients will have large-vessel occlusion in the M1 distribution (1). The advent of endovascular mechanical thrombectomy has drastically improved the morbidity and mortality associated with large-vessel occlusions when compared with intravenous tissue plasminogen activator (tPA) alone (2). Indeed, thrombectomy is considered the first-line therapy for patients with internal carotid artery and M1 occlusions presenting within 6 h of stroke onset (2, 3). Furthermore, recent recommendations have resulted in the expansion of thrombectomy indications to include patients with M2 and M3 occlusions and large-vessel posterior circulation occlusions, as well as increasing the time window for intervention in patients with large-vessel anterior circulation occlusions. Although this has led to improved clinical outcomes overall, despite aggressive intervention many patients are left severely disabled and die (4, 5). The ability to predict which patients die after thrombectomy will be an important aspect of clinical decision making, enhance family discussions, and improve resource management. Here we discuss our institution's experience with patient deaths after endovascular mechanical thrombectomy for the treatment of acute ischemic stroke and evaluate which clinical variables can be predictive of this outcome.

## Materials and Methods

We retrospectively reviewed the charts of all patients who underwent mechanical thrombectomy at our institution between January 2012 and January 2017 who had full code status at the time of treatment. The Institutional Review Board (IRB #00115230) approved this study with a waiver of informed consent to ensure comprehensive inclusion of patients. Relevant patient demographic data, National Institutes of Health Stroke Scale (NIHSS) score, past medical history, and treatment details (including hospital transfers, time to revascularization, length of procedure, Thrombolysis in Cerebral Infarction [TICI] score, and intraprocedural complications) were collected. The dichotomous patient outcome (i.e., alive or dead) at 90 days was also recorded. All patient information was de-identified and analyzed in compliance with Health Insurance Portability and Accountability Act regulations. Patients were excluded from the study if data was incomplete or missing. Thrombectomy was indicated in patients 1) if their presentation was within 6 h of symptom onset, or 2) if their presentation was within 6–24 h while their ASPECT score was > 6 and/or CTP showed a mismatch with penumbra. We compared patient data based on outcome via independent sample *t*-test and Pearson's and Fisher's chi-squared test depending on variable type to identify any identifiable risk factors associated with death after thrombectomy. Significance was defined *a priori* as a *p* < 0.05. Statistically significant risk factors were then analyzed in a univariate analysis followed by a subsequent multivariate analysis. All statistical tests were completed using IBM SPSS Statistics (V20.0, IBM, Armonk, NY).

## Results

During the study period, 134 patients underwent mechanical thrombectomy; sufficient clinical data was available in 111 of the patients. The patient demographic and post-procedural data are summarized in Table 1. Among the included patients, 42 patients who underwent thrombectomy died during the 90 day post-procedural period; 69 patients survived this period. There were no intraprocedural deaths. Patients who survived were not significantly younger than those who died (60.4 ± 17.3 years vs. 66.6 ± 18.3 years, *p* < 0.076). The rates of common comorbid medical conditions such as hypertension, hyperlipidemia, coronary artery disease, atrial fibrillation, or a history of prior stroke or transient ischemic attack were not significantly different between the two groups. The mean NIHSS score at presentation was 14.9 in surviving patients and 19.6 in non-surviving patients (*p* < 0.002). Surviving patients were also less likely to have a history of cancer (4.4% vs. 26.2%, *p* < 0.002). TICI 2b/3 revascularization was more common among survivors (78.3% vs. 50.0%, *p* < 0.003), and they experienced fewer intra-procedural technical complications (7.4% vs. 26.2%, *p* < 0.01). Overall, there were 16 complications (1 intraprocedure thrombus, 1 patient-pulled access catheter, 3 vessel perforations, 5 vessel dissections, 6 post-procedural groin hematomas). There was no difference in survival status related to whether patients underwent a decompressive hemicraniectomy or to the location of their stroke (anterior circulation vs. posterior) (10 posterior strokes in survivor group (14.5%) vs. 9 posterior strokes in non-surviving group (21.4%), *p* = 0.347). Finally, survivors were less likely to experience hemorrhagic conversion (21.7% vs. 47.6%, *p* < 0.004).

**Table 1:** Summary of demographic and procedural data.

**Variable**	**Patients who survived (*n =* 69)**	**Patients who died (*n =* 42)**	***P*-value**
Age (years)	60.4 ± 17.3	66.6 ± 18.3	0.076
Sex, male	30 (43.5%)	14 (33.3%)	0.289
NIHSS score	14.9 ± 7.1	19.6 ± 8.3	**0.002**
Transferred from an outside hospital	38 (55.1%)	24 (57.1%)	0.831
Number of co-morbidities	2.3 ± 1.7	2.5 ± 1.5	0.689
Hypertension	43 (62.3%)	29 (69.0%)	0.471
CAD	17 (24.6%)	9 (21.4%)	0.699
DM	20 (29.0%)	12 (28.6%)	0.963
HLD	37 (53.6%)	18 (42.9%)	0.271
Afib	20 (29.0%)	8 (19.0%)	0.242
History of cancer	3 (4.4%)	11 (26.2%)	**0.002**
Hx of prior DVT	2 (2.9%)	6 (14.3%)	0.056
Hx of prior stroke	20 (29.0%)	11 (26.2%)	0.753
New STEMI during admission	4 (5.8%)	7 (16.7%)	0.098
Time to revascularization (min)	348.4 ± 270.7	398.6 ± 295.0	0.560
Procedure length (min)	73.2 ± 38.3	87.1 ± 46.4	0.120
Hemorrhagic conversion	15 (21.7%)	20 (47.6%)	**0.004**
Procedural complications	5 (7.4%)	11 (26.2%)	**0.011**
TICI 2b or 3 revascularization	54 (78.3%)	21 (50.0%)	**0.003**
Treated with tPA and thrombectomy	46 (66.7%)	29 (69.0%)	0.797
Decompressive hemicraniotomy	4 (5.80%)	1(2.38%)	0.648
Location of stroke, posterior	10 (14.5%)	9 (21.4%)	0.347

*Data are reported as mean ± standard deviation or total (%) as appropriate*.

*Boldface values are statistically significant at p < 0.05*.

*NIHSS, National Institutes of Health Stroke Scale; CAD, coronary artery disease; DM, diabetes mellitus, HLD, hyperlipidemia; Afib, atrial fibrillation; DVT, deep vein thrombosis; STEMI, ST-elevation myocardial infarction; TICI, thrombolysis in cerebral infarction; tPA, tissue plasminogen activator*.

Because presenting NIHSS score, a history of cancer, hemorrhagic conversion, and technical complications were statistically different between the two groups, they were further evaluated in univariate and multivariate analyses (Table 2). Revascularization was not evaluated in this manner because it represented a treatment outcome. Each of these variables retained statistical significance in multivariate analysis, indicating that they were independent predictors of death after thrombectomy. Of the variables included in the multivariate analysis, a history of cancer was the strongest indicator of the risk of death after thrombectomy (OR 22.94, CI 4.03–130.35, *p* < 0.001).

**Table 2:** Univariate and multivariate analyses.

**Variable**	**Univariate analysis**			**Multivariate analysis**		
	**Odds ratio**	**95% CI**	***P*-value**	**Odds ratio**	**95% CI**	***P*-value**
Cancer	7.80	2.03–29.99	0.003	22.94	4.03–130.35	<0.001
NIHSS score	1.08	1.02–1.14	0.004	1.098	1.03–1.17	0.004
Hemorrhagic conversion	3.27	1.42–7.52	0.005	2.78	1.00–7.67	0.048
Procedural complication	4.47	1.42–13.99	0.010	4.50	1.15–17.63	0.031

*CI, confidence interval; NIHSS, National Institutes of Health Stroke Scale*.

## Discussion

In recent years, the effectiveness of mechanical thrombectomy over intravenous tPA alone has become undoubtedly clear in improving outcomes among patients with large-vessel occlusions (2, 4, 6–8). The recent publication of the DEFUSE-3 and DAWN trials and the consequent expansion of the time window for thrombectomy further revolutionized the indications for endovascular stroke intervention. The potential for additional expansion of thrombectomy indication criteria to include patients with large core infarcts at baseline and those with low presenting NIHSS (9–12), as well as improving patient access to thrombectomy at various hospitals represent key drivers for exponential growth in endovascular stroke treatment. However, despite the widespread use of advanced imaging techniques and vetting of patients for suitability for endovascular thrombectomy, a significant number of patients remain neurologically devastated and often progress to death. In our experience, this outcome can occur in as many as 38% (42/111) of patients within 90 days after thrombectomy for both anterior and posterior circulation large-vessel occlusions cases. Similarly, others have reported death rates that range from 22 to 39% with variations owing to study population size, follow-up periods, and inclusion criteria (13–15).

The cause of death after thrombectomy remains poorly understood but important when selecting patients for the procedure. In our study, we evaluated risk factors associated with stroke (hypertension, hyperlipidemia, coronary artery disease, atrial fibrillation) and found no significant difference in the prevalence of these pre-morbid risk factors between patients who survived after thrombectomy and non-survivors. Fonarow et al. (16) demonstrated that presenting NIHSS was a predictor of mortality at 30 days in patients with acute infarcts, with an NIHSS score above 17 conferring an 86% risk of death in comparison with an NIHSS score of 4 being associated with a 14% risk of dying. In our treated population, although there was a significant difference in NIHSS scores between survivors and those that did not, a higher NIHSS score was found to minimally increase the risk of mortality (OR 1.098, CI 1.03–1.17, *p* < 0.004) in a multivariate analysis.

A known complication of ischemic infarcts is hemorrhagic conversions, which can occur in as many as 30% of patients after treatment with tPA. This risk seems to be increased after endovascular interventions, but the rate of symptomatic hemorrhagic conversions appears to be comparable with that of medical treatment alone (17). In our cohort of treated patients, 32% experienced a hemorrhagic conversion after thrombectomy, which was associated with a significant increase in the risk of death (OR 2.78, CI 1.00–7.67, *p* < 0.048). Both subarachnoid and intraparenchymal hemorrhage can occur as a result of endovascular treatments. Parenchymal hemorrhages generally result from reperfusion injury, which is generally thought to be related to necrosis of the vessel wall due to prolonged ischemia. Additionally, hemorrhage can occur because of direct vessel wall perforations, which are a known complication of endovascular thrombectomy. Technical complications, including 3 vessel perforations resulting in 2 intraparenchymal hemorrhages and 1 patient with a small amount of subarachnoid hemorrhage, did contribute to an increased risk of death (OR 4.50, CI 1.15–17.63, *p* < 0.031) in our treatment group. Vessel wall injuries generally result while crossing the lesion because the distal vessel is not visible during this time (18). Importantly, all 3 patients that experienced vessel wall perforation died during the 90 day post-procedural period.

Of the comorbidities analyzed, pre-morbid cancer diagnosis was the most significant risk factor associated with death after thrombectomy. Patients with a history of cancer were found to have a 22-fold increase in risk of death (OR 22.94, CI 4.03–130.35) during the 90 day period after thrombectomy. This a substantial increase in risk compared with cancer alone, which is thought to contribute a 10- to 12-fold increase in risk of death (19). Additionally, cancer patients have increased rates of depression, which worsens their morbidity and increases healthcare cost (20).

In general, deaths after acute infarct are associated with the degree of neurologic impairment and the number of patient comorbidities (21). Over 50% of our treated patients were transferred from outside hospitals, and the average time to revascularization was close to 4.5 h. Although these variables were not identified as being associated with an increased risk of death in our study, this additional delay in care likely contributes to worsening neurologic injury and reduces the degree of neurologic improvement after thrombectomy (5). Previous larger studies have demonstrated the importance of time to revascularization; our cohort was likely too small to capture this difference, but the initial effect of the presenting patient NIHSS demonstrates the importance of treating acute infarcts as quickly as possible to prevent unrecoverable ongoing injury (5, 22). Taken together, these overwhelming circumstances likely all play a role in patients succumbing to their disease despite intervention.

## Limitations

The findings of our study reveal important associations; however, limitations should be considered. Our study represents a single institutional experience and is limited by its retrospective design. The strong association with cancer could be confounded by the many medical conditions associated with cancer. Although we evaluated common comorbidities including DVTs and PEs, other factors including immunodeficiency and overall health status related to chemotherapy and radiation treatment are difficult to account for. Additionally, our small patient sample size limits the generality of our analysis.

## Conclusion

As we continue to push the envelope to increase the allowable time for thrombectomy after ischemic cerebral infarct, we will face the dilemma of making the decision to treat a patient in the setting of significant comorbidities. Although advances in endovascular treatments continue to improve, death after thrombectomy remains the outcome for a considerable number of patients. Death appears to be more prevalent among patients with a history of cancer, and thus a thorough discussion of expected outcomes is needed to avoid unnecessary medical cost, patient morbidity, family grief, and efficient use of a medical resources.

## Data Availability Statement

The datasets generated for this study are available on request to the corresponding author.

## Ethics Statement

The studies involving human participants were reviewed and approved by University of Utah IRB. Written informed consent for participation was not required for this study in accordance with the national legislation and the institutional requirements.

## Author Contributions

A-WA collected data, completed statistical calculations, and wrote the manuscript. CK assisted in study design and revised the manuscript. VR assisted with statistical calculations and revised the manuscript. JS and EJ collected data. RG revised the manuscript. PT designed the study and revised the manuscript.

### Conflict of Interest

PT is a consultant for Medtronic, Stryker Neurovascular, and Cerenovus. The remaining authors declare that the research was conducted in the absence of any commercial or financial relationships that could be construed as a potential conflict of interest.
